# Enrichment of Murine CD68^+^CCR2^+^ and CD68^+^CD206^+^ Lung Macrophages in Acute Pancreatitis-Associated Acute Lung Injury

**DOI:** 10.1371/journal.pone.0042654

**Published:** 2012-10-22

**Authors:** Hamid Akbarshahi, Mandy Menzel, Monika Posaric Bauden, Ann Rosendahl, Roland Andersson

**Affiliations:** 1 Department of Surgery, Clinical Sciences Lund, Lund University, Lund, Sweden; 2 Department of Surgery, Clinical Sciences Lund, Skåne University Hospital and Lund University, Lund, Sweden; Gentofte University Hospital, Denmark

## Abstract

Acute lung injury (ALI) is an important cause of mortality in critically ill patients. Acute pancreatitis (AP) is one of the risk factors for developing this syndrome. Among the inflammatory cells, macrophages have a key role in determining the severity of the acute lung injury. In the lungs, macrophages constitute a heterogeneous cell population distributed in different compartments. Changes in not only the macrophage count, but also in their phenotype have been seen during the course of lung injury. A murine ductal ligation model of acute pancreatitis showed substantial morphological changes in the pancreas and lungs. Immunohistochemistry showed neutrophil recruitment into both organs after 9 hours and later on. F4/80^+^ cells in the pancreas increased in the ligated animals, though there was not a significant difference in their number in the lungs as compared to sham operated animals. Flow cytometry analysis of lung macrophages demonstrated an enrichment of F4/80^−^ CD68^+^CCR2^+^ and F4/80^−^ CD68^+^CD206^+^ lung macrophages in ligated animals (AP) as compared to the sham operated group. The level of interleukin-6 in plasma increased 3 hours after ligation compared to the sham operated group, as a first indicator of a systemic inflammatory response.

This study suggests a role for F4/80^−^ CD68^+^ macrophages in the pathogenesis of acute lung injury in acute pancreatitis. Studying lung macrophages for different phenotypic markers, their polarization, activation and recruitment, in the context of acute lung injury, is a novel area to potentially identify interventions which may improve the outcome of acute lung injury.

## Introduction

The incidence of acute pancreatitis has been reported to be increased during the last two decades [1]. In 80% of patients, the acute pancreatitis is considered as mild and resolves without serious morbidity. However, up to 20% of patients develop a severe disease with local pancreatic and extra-pancreatic complications [2]. Gallstone disease and alcohol abuse are the most frequent causes of acute pancreatitis in adults [3]. Treatment of mild disease is supportive, while severe episodes require management by a multidisciplinary team. The incidence of pulmonary complications is high in severe pancreatitis, ranging from 15 to 55%, and the severity of pulmonary complications can vary widely from mild hypoxemia without clinical or radiological abnormalities, to the severe acute respiratory distress syndrome [4]. The underlying mechanisms involved in the pathogenesis of acute pancreatitis-induced acute lung injury (ALI) are poorly understood. Current treatment options are limited, and predominantly aimed at supportive therapy.

Although neutrophil recruitment into the lungs is a hallmark of ALI, macrophages, which reside in the pulmonary interstitium and alveoli, are key effector cells of the inflammatory response. Macrophages have both pro- and anti-inflammatory phenotypes. However, these phenotypes have been defined predominantly in *in vitro* cultures of macrophages and it is largely unknown how these diverse phenotypes of macrophages contribute to different types of tissue injury *in vivo* [5].

Pulmonary macrophages do not remain committed to a single activation profile which determines whether lung tissue will face destruction or recovery. Functionally, distinct subsets of macrophages may exist in the same tissue and play critical roles in both initiation and recovery of inflammation. Therefore, the origin and activation state of the macrophages and the microenvironment in which they reside, are critical determinants of their response to lung injury. The heterogeneity of macrophages, their diverse role in pulmonary inflammation and tissue remodeling, and the coordinated activation and programming by other inflammatory and parenchymal cells are not fully understood. However, it becomes increasingly evident that cross-talk of various signals at different levels influences on the generation of functional macrophage programs, with a variety of signals being integrated to shape a distinct phenotype at a defined stage of inflammation [6].

Chemokine (C-C motif) receptor 2 (CCR2) and its major ligand, Chemokine (C-C motif) ligand 2 (CCL2), are evidently important in both emigration of these cells from the bone marrow into the blood stream and their immigration into inflamed tissues, where they undergo differentiation and polarization into macrophages that can be categorized as either classically activated (M1) or alternatively activated (M2) [7], [8], [9].

A better understanding of the underlying pathophysiology of severe acute pancreatitis-induced ALI may lead to more targeted therapeutic options, potentially leading to improved survival. Animal models of acute pancreatitis are therefore an essential investigative tool for these aims.

In the present study, we have studied the dynamics of macrophages in lung tissue in a murine model of acute pancreatitis-associated acute lung injury.

## Materials and Methods

### Animals

8–10 week old male wild-type C57BL/6 mice were purchased from Charles River, Germany. The mice were housed in appropriate facilities at Lund University, under specific pathogen-free conditions and handled according to the institute guidelines with approval of the Malmo-Lund Animal Care Ethics Committee (M263-10). The animals were kept under 12/12 h light/dark regime in standard mesh cages with laboratory chow and drinking water ad libitum.

### Animal model

Acute pancreatitis was induced using the combined pancreatic duct and bile duct (BPD) ligation model as described by Samuel *et al* [10]. Briefly, the mice were anesthetized and maintained with 2–4% isoflurane. Under aseptic conditions, a midline laparotomy was performed. The bile duct, proximal to its entry into the pancreas, and the common bile-pancreatic duct, near its junction with the duodenum, were dissected and ligated (BPD group). The same procedure was applied to sham-operated control mice where the common bile-pancreatic duct and the bile duct were dissected, but not ligated, after which the abdomen was closed. The mice recovered rapidly after surgery and postoperative buprenorphine analgesia (0.05 mg/kg, s.c.) was administered twice daily. The animals (n = 10 in each group) were sacrificed by exsanguination through puncture of the abdominal aorta 1, 3, 9, 24 and 48 h after pancreatitis-induced surgery and plasma samples were collected and stored at −80°C until analysis. The right ventricular cavity was cannulated and perfused with 5 ml EDTA PBS. Biopsies of the pancreatic duodenal lobe and lungs were harvested, immediately processed for flow cytometry evaluation or snap-frozen in liquid nitrogen and stored at −80°C until analysis. For histological and immune-staining, the samples were fixed in 4% paraformaldehyde.

### Immunohistochemistry

Paraffin embedded tissues were sectioned 5 µm thick and processed routinely for hematoxylin and eosin (H&E) staining. For immunohistochemistry, the sections were exposed to H_2_O_2_/methanol, levamisol (DakoCytomation) and avidin/biotin blocking (Vector Laboratories) to neutralize endogenous peroxidase, phosphatase and biotin, respectively. Sections were probed with antibodies against either neutrophils (1∶300, NIMP-R14, Abcam, Cambridge, UK) or F4/80 (1∶500, MCA497GA, AbD Serotec, Oxford, UK) and CD68 (1∶50, MCA1957B, AbD Serotec, Oxford, UK) for monocytes/macrophages. The sections were then incubated with appropriate biotinylated secondary antibodies (1∶200, ABC Vectastain, Vector Laboratories, Burlingame, CA, USA) in 2% normal blocking serum and visualized using 3, 3′-diaminobenzidine (DAB, Vectastain, Vector Laboratories). The sections were analyzed using a Nikon Eclipse E800 microscope, Olympus DP70 camera and Nis elements software. The total number of neutrophils and monocytes in the entire sections were calculated and the total parenchymal area was measured using ImageJ 1.44 (National Institute of Health, USA). Inflammatory cell counts were quantified as total number of cells per mm^2^ tissue.

### Myeloperoxidase activity

Myeloperoxidase (MPO) activity was measured according to Koike *et al.* [11], with minor modifications. Cryopreserved tissue samples were homogenized and washed in gradually increasing concentrations of PBS. The supernatants were incubated with 3,3′,5,5′-tetramethylbenzidine for 2 minutes in the presence of hydrogen peroxide (H_2_O_2_) and the reaction subsequently stopped by the addition of sulphuric acid (2M, H_2_SO_4_). The samples were measured on a Labsystems Multiskan Plus plate reader (test wavelength 450 nm, reference wavelength 540 nm) using the DeltaSoft JV software (BioMetallics Inc., Princeton, NJ) with horseradish peroxidase as standard.

### Flow cytometric analysis

Single-cell lung suspensions were prepared from mice sacrificed at 9 and 24 h. Briefly, the right lung was removed, minced on ice and digested in RPMI 1640 containing 1.33 mg/ml collagenase (Roche Diagnostics GmbH, Penzberg, Germany) and 0.1 kU/ml DNase (Sigma-Aldrich, St. Louis, MO, USA) at 37°C for 60 min. The digested lung tissue was filtered through a 70-µm sieve, the total cell number counted and non-specific binding to Fc Receptors blocked using anti-CD16/CD32 antibodies. The single-cell suspensions were stained with antibodies specific for CD11c (BD Biosciences, San Jose, CA, USA), CCR2 (R&D Systems, Minneapolis, MN, USA) and F4/80 (Biolegend, San Diego, CA, USA), then fixed and permeabilized with Cytofix-Cytoperm solution (BD Biosciences) and subsequently stained with anti-CD68 and anti-CD206 (Biolegend, San Diego, CA, USA) antibodies. Approximately 2×10^5^ events (cells) were collected for each sample on a FACSCalibur (Becton Dickinson), dual laser, flow cytometer using CellQuest Pro Software (BD Biosciences), and analyzed using FlowJo software (Tree Star Inc, CA, USA).

### Cytokine measurement

Cryopreserved pancreatic and lung tissues were homogenized in 20 mM HEPES buffer (pH 7.4) supplemented with 1.5 mM EDTA and protease inhibitors (Complete, Roche Diagnostics GmbH, Mannheim, Germany). Local pancreatic and lung CXCL1 and CCL2 levels were assessed in duplicates using enzyme-linked immunosorbent assays (ELISA) according to the manufacturer's instructions (R&D Systems, Minneapolis, MN, USA).

Systemic cytokine levels were measured in plasma using MSD mouse proinflammatory 7-plex ultra-sensitive assay (Mesoscale Discovery, Gaithersburg, MD, USA) according to the manufacturer's instructions. The lower level of detection and coefficient variation (CV) range for seven analytes were: IL-6 (4.5 pg/ml, 2.8–8.6%), IL-10 (11 pg/ml, 1.1–5.8%), tumor necrosis factor (TNF)-α (0.85 pg/ml, 1.9–9%), IL-1β (0.75 pg/ml, 1.8–8.4%), IL-12p70 (35 pg/ml, 1.1–8.2%), IFN-γ (0.38 pg/ml, 1–7.3%) and CXCL1 (3.3 pg/ml, 2.8–8.3%), respectively. In the present study, the average CVs for IL-6, CXCL1 and IL-10 were 6%, 9% and 13%, respectively. The average CVs for TNF-α in the analytes with the mean concentration over the threshold of 5 pg/ml was 7.3%.

### Statistics

The data is presented as mean ± SEM. Statistical significance was determined by two-tailed student t-test using Prism software. A *P*-value of <0.05 was considered statistically significant.

## Results

### Morphological and physiological changes

**Figure 1:**
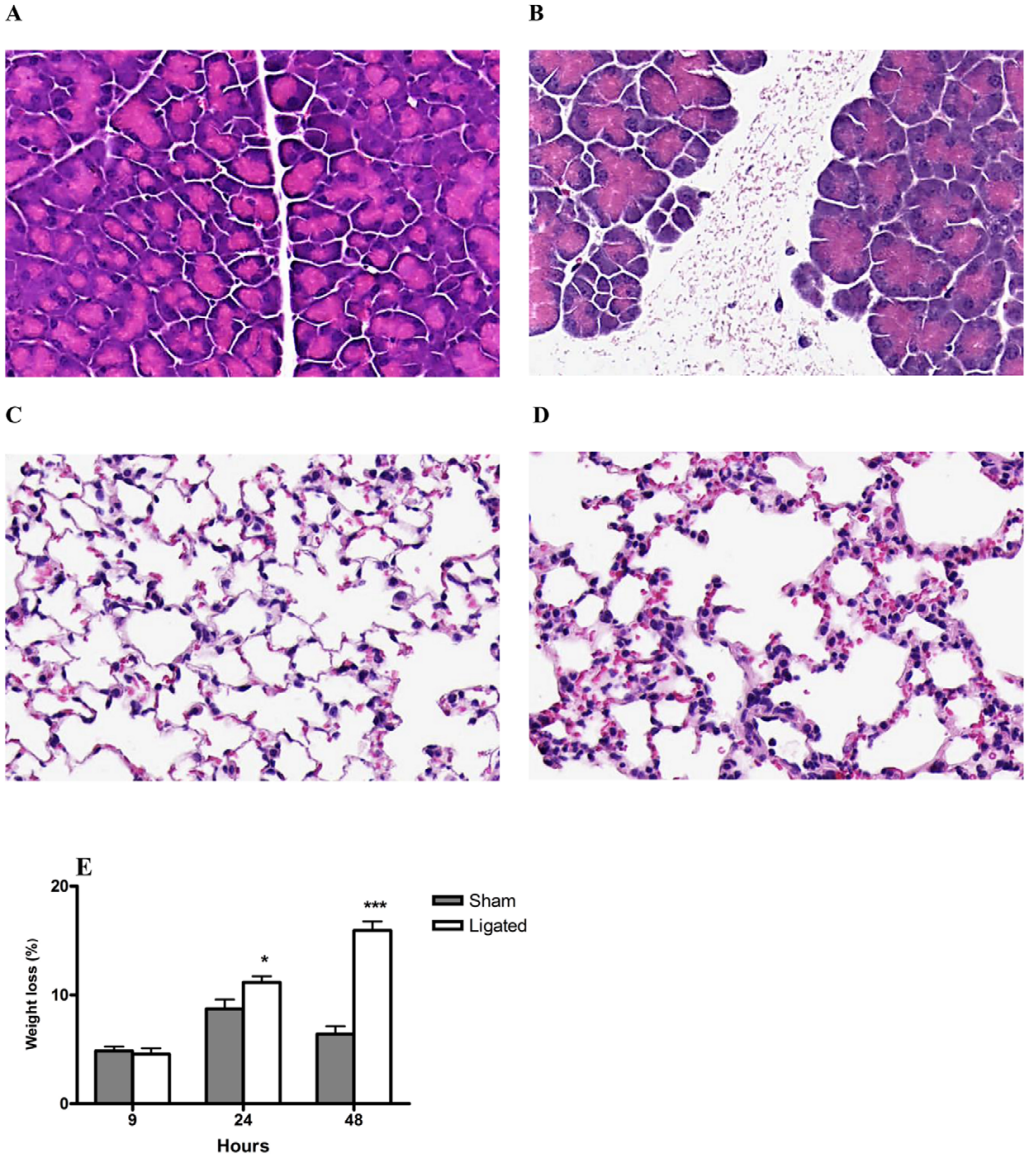
Morphological and physiological changes associated with acute pancreatitis and associated acute lung injury. Representative H&E images of pancreas (A, B) and lungs (C, D) 24 hours after induction of pancreatitis through BPD ligation (A, C) are depicted. Pronounced edema and necrosis observed in the pancreas after pancreatitis induction (B) compared to sham control (A). Lung section shows thickening of alveolar septa, hemorrhage and edema after pancreatitis induction (D) compared to sham control (C). Original magnification, 20×. Mice experienced a significant weight loss 24 and 48 h after induction of pancreatitis through BPD ligation compared to sham control (E). Data expressed as mean ± SEM, n = 10 per group. *P<0.05, ***P<0.001 versus sham control, by two-tailed Student *t*-test.

**Figure 2:**
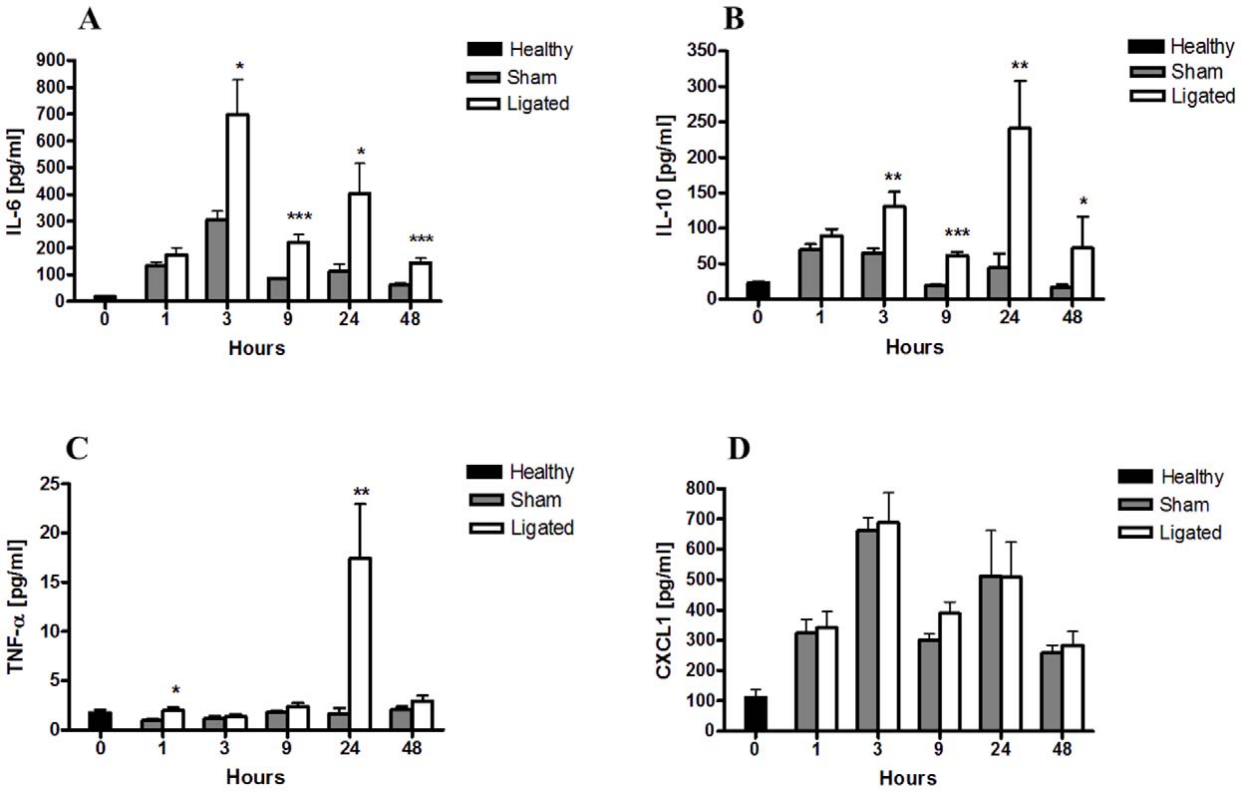
Acute pancreatitis induces changes in systemic inflammatory cytokine levels. Changes in plasma IL-6 (A), IL-10 (B), TNF-α (C) and CXCL1 (D) levels at the indicated time points were evaluated by MSD multiplex assay. Pancreatitis induced an early systemic rise in IL-6 and IL-10 levels that remained significant throughout the study. TNF-α plasma level peaked at 24 h, while no significant difference in CXCL1 plasma levels between pancreatitis and sham control was observed at any time point studied (D). Bars show mean ± SEM, n = 10 per group. *P<0.05, **P<0.01, ***P<0.001 versus sham control, by two-tailed Student *t*-test.

Acute pancreatitis was induced using a BPD ligation model to mimic gallstone-induced acute pancreatitis. Characteristic morphological features associated with acute pancreatitis, edema and necrosis were observed in the pancreas from 9 h after induction, with increasing severity after 24 h (Figure 1B) and 48 h. The induced acute pancreatitis resulted in a rapid onset of acute lung injury. Morphological changes in the lungs with alveolar septal thickening, hemorrhage, edema and vascular congestion were observed from 9 h post pancreatitis induction and were further exacerbated at 24 h (Figure 2D) and at 48 h. There were no morphological changes observed in the pancreas (Figure 1A) or the lungs (Figure 1C) in the sham operated animals at any time point studied.

The severity of the induced acute pancreatitis and the associated lung injury was further manifested by a significant weight loss in the mice over time. A 16% weight loss was observed in the pancreatitis group after 48 h compared to 5% in the sham group (*P*<0.001; Figure 1E).

### The earliest differences in plasma levels of pro-inflammatory cytokines were seen after 3 hours

When evaluating the systemic inflammatory response following pancreatitis induction, we found both IL-6 and IL-10 plasma levels to be significantly elevated in the BPD-ligated animals at 3 h and these were still enhanced at 48 h. While plasma IL-6 levels strongly peaked at 3 h (Figure 2A), IL-10 further increased to reach maximum levels 24 h after pancreatitis induction (Figure 2B). A pronounced TNF-α peak was present in at 24 h, while at 48 h after pancreatitis induction this expression returned to control values (Figure 2C). In contrast, systemic CXCL1 levels did not significantly differ between the ligated and the sham treated animals at any time point (Figure 2D).

### Elevated local pancreatic and lung CXCL1 and CCL2 levels in BPD-ligated mice

**Figure 3:**
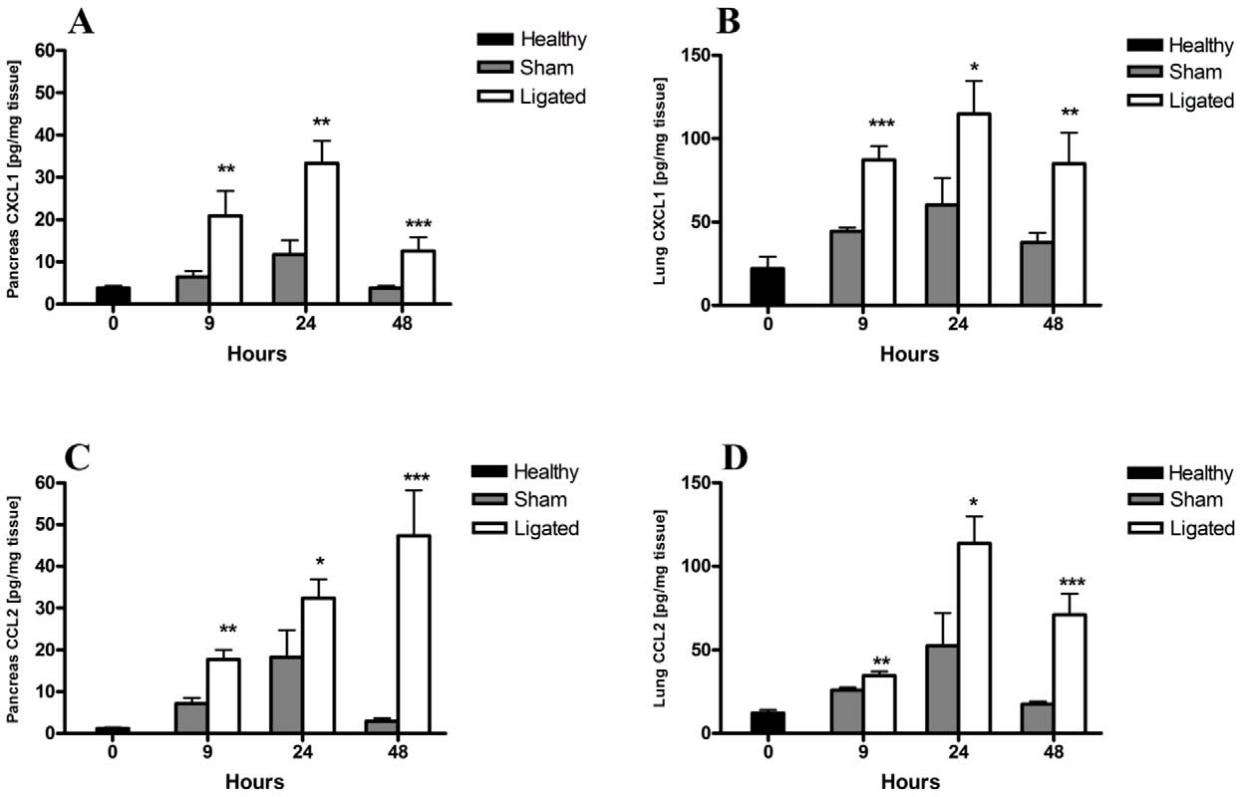
Acute pancreatitis induces changes in local pancreatic and lung chemokine levels. Tissue homogenates from pancreas (A, C) and lungs (B, D) were evaluated by ELISA for CXCL1 (A, B) and CCL2 (C, D) levels at the indicated time points. Both CXCL1 and CCL2 levels were significantly elevated in the pancreas (A) and lungs (B) 9, 24 and 48 h after pancreatitis induction compared to sham controls. Bars show mean ± SEM, n = 10 per group. *P<0.05, **P<0.01, ***P<0.001 versus sham control, by two-tailed Student *t*-test.

To determine the expression of local soluble inflammatory mediators, IL-6, Chemokine (C-X-C motif) ligand 1 (CXCL1) and CCL2 levels in pancreatic and lung homogenates were evaluated. In contrast to the systemic rise in IL-6 levels found, no difference in the local pancreatic or lung IL-6 levels were observed between the ligated and sham operated group at any time point (data not shown). However, levels of the neutrophil chemoattractant CXCL1 increased approximately 2-fold in both the pancreas and lungs 9 h after pancreatitis induction as compared to controls peaked at 24 h and then declined (Figures 3A and B). Similarly, levels of the monocyte chemoattractant CCL2 were significantly elevated 9 hours after pancreatitis induction in both pancreas and lungs. The CCL2 levels increased over time in the pancreas following ligation, while peaking at 24 h in the lungs (Figures 3C and D). Induction of the inflammatory mediators CXCL1 and CCL2 in both pancreas and lungs suggest the involvement of neutrophils and monocytes/macrophages in both tissues.

### Neutrophil recruitment into the pancreas and lungs following acute pancreatitis induction

**Figure 4:**
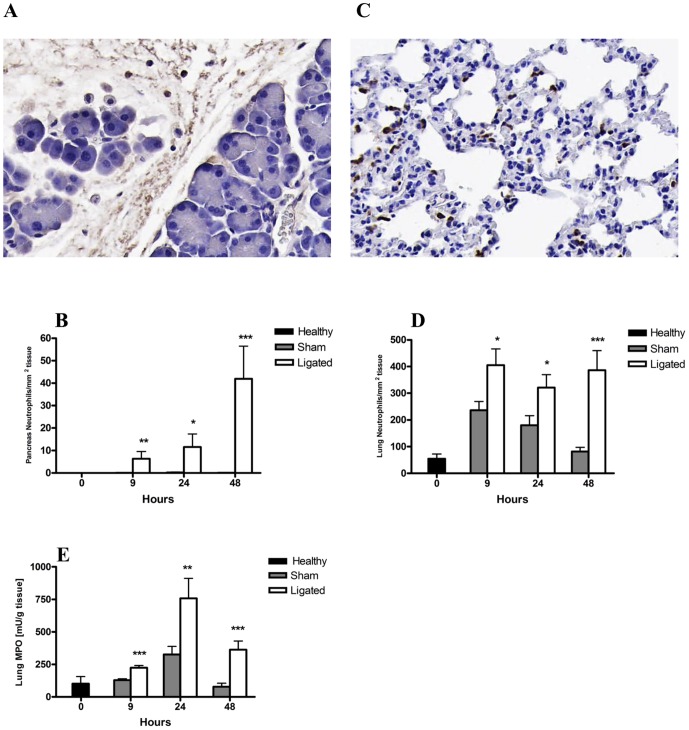
Changes in pancreatic and lung neutrophil number during acute pancreatitis. Representative photomicrographs of pancreas (A) and lung (C) tissue stained for NIMP-R14, showing infiltration of neutrophils (brown) 24 h after induction of pancreatitis (original magnification, ×20). The number of NIMP-R14^+^ neutrophils in the pancreas (B) and lungs (D) were significantly enriched following pancreatitis induction (ligated) compared to sham control. Lung MPO activity was significantly increased following acute pancreatitis compared to sham controls at all time points evaluated (Fig. E). Bars show mean ± SEM, n = 10 per group. *P<0.05, **P<0.01, ***P<0.001 versus sham control, by two-tailed Student *t*-test.

The inflammatory response following pancreatitis induction was marked by the recruitment of neutrophils (NIMP-R14 positive cells) into both the pancreas (Figure 4A) and lungs (Figure 4B). A significant neutrophil infiltration was evident in the pancreas already 9 h after acute pancreatitis induction compared to sham operated mice, and the number gradually increased to reach approximately 40 neutrophils/mm^2^ at 48 h compared with <1 neutrophil/mm2 found in the sham controls (*P*<0.001; Figure 4C).

In addition to local pancreatic neutrophil recruitment, a significant neutrophil infiltration into the lungs was observed from 9 h post pancreatitis induction as compared to sham controls. In the lungs, the neutrophil levels stabilized at the high 400 cells/mm^2^ by 9 h and no further increase was observed at later time points (Figure 4D). Lung MPO levels, as an indicator of neutrophil activity, confirmed the significant neutrophil infiltration in the lungs from 9 h, with a peak activity noted at 24 h after pancreatitis induction (Figure 4E). The levels of MPO activity in the pancreas were below the assay detection limit.

### F4/80 expressing cells were recruited into the pancreas following ligation

**Figure 5:**
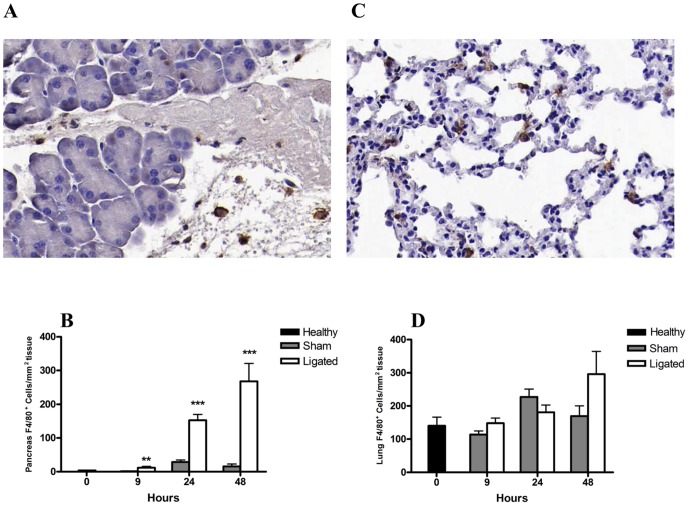
Changes in pancreatic and lung F4/80^+^ macrophage number during acute pancreatitis. Representative photomicrographs of pancreas (A) and lung (C) sections stained for F4/80 as indicator of macrophage infiltration (brown) 24 h after pancreatitis induction (original magnification, ×20). Quantification of F4/80^+^ cells showed a significantly increased enrichment in the pancreas from 9 h following pancreatitis compared to sham controls (B). No significant difference was observed in the number of lung F4/80^+^ cells between pancreatitis and sham control groups (D). Bars show mean ± SEM, n = 10 per group. **P<0.01, ***P<0.001, by two-tailed Student *t*-test.

The acute pancreatitis following BPD-ligation was further associated with a significant increase in macrophage recruitment into the pancreas as measured by F4/80 positive cells (Figure 5A). The F4/80^+^ macrophage infiltration was evident from 9 h and continued to a 17-fold increase by 48 h compared to sham operated mice (*P*<0.001; Figure 5B). In contrast, no significant recruitment of F4/80^+^ macrophages was found in the lungs (Figure 5C), although a non-significant trend to raised F4/80^+^ macrophage count was noted at 48 h (*P* = 0.07; Figure 5D).

### CD68^+^ F4/80^−^ cells increased in the lungs in ligated mice

**Figure 6:**
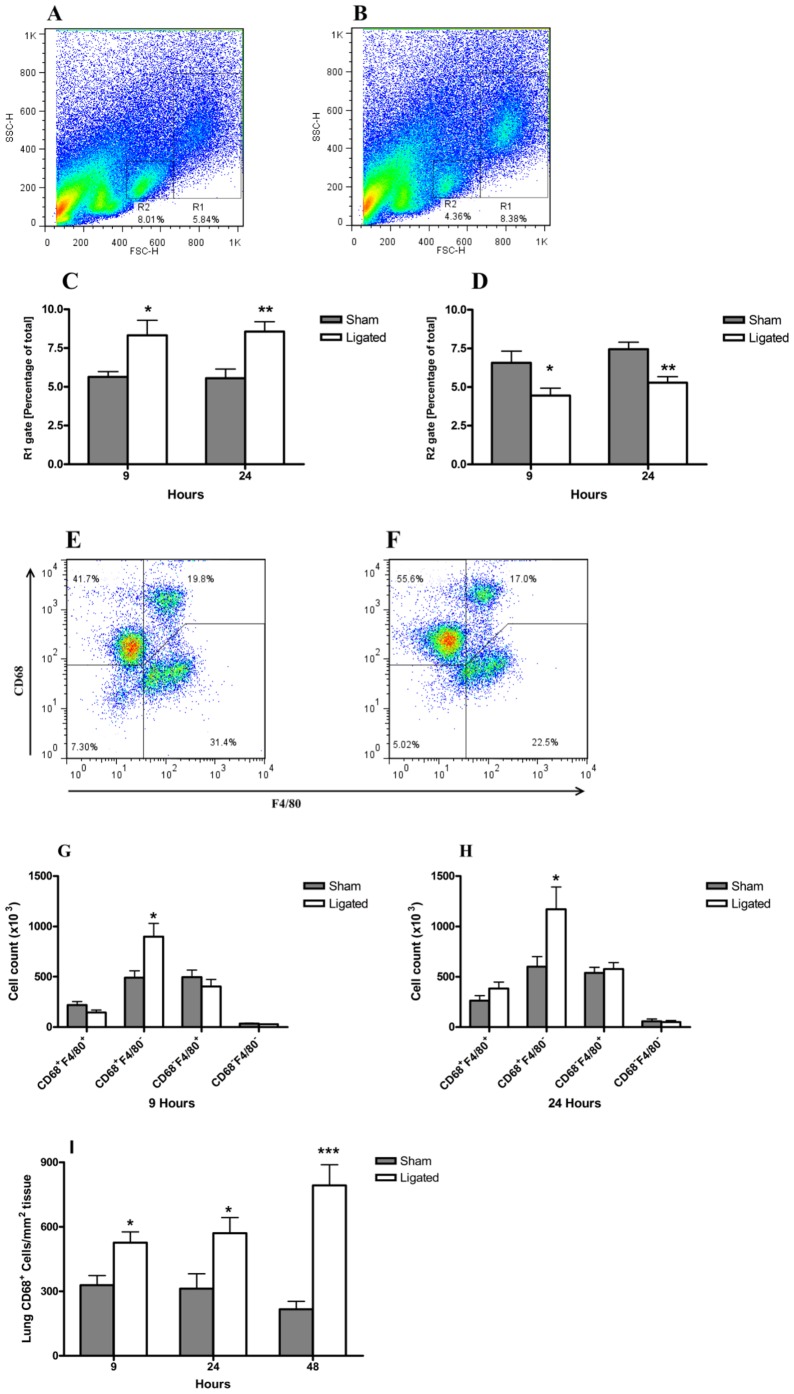
Changes in lung macrophage sub-populations during acute pancreatitis. Single cell preparations of the right lung were evaluated by flow cytometry. Dot plots from one representative experiment of sham control (A) and 24 h post pancreatitis induction (B) showing the gating strategy. Significant modulations in the percentage of R1 (C) and R2 (D) gated populations following acute pancreatitis compared to sham operated animals. Representative profiles of CD68 and F4/80 expressing cells in the R1 population of sham (D) and ligated (E) mice after 24 h are shown. A significant enrichment in the total number of R1 gated CD68^+^ F4/80^−^ cells in the right lung 9 h (F) and 24 h (G) after pancreatitis induction compared to sham controls. CD68^+^ cells were increased significantly in the immunohistochemical staining of the lung sections in the acute pancreatitis compared to sham at 9, 24 and 48 h. , n = 8 per group. *P<0.05, **P<0.01, ***P<0.001 versus control, by two-tailed Student *t*-test.

To further characterize the macrophage population present in the lungs following pancreatitis induction, flow cytometry analysis was applied on the single cell preparation of the lungs. Two distinct cell populations, as defined by their size (FSC-H) and granularity (SSC-H), were markedly modulated in the pancreatitis group at 9 and 24 h (Figures 6A and C) compared to sham controls (Figures 6B and D). While a significant increase in cell number in the R1 gated population was observed (Figure 6C), a concomitant and significant decrease in the R2 gated population occurred (Figure 6D). Further evaluation of the populations revealed that the increased R1 population was related to a two-fold enrichment of CD68^+^F4/80^−^ cells at both 9 and 24 h compared to sham control mice (P<0.001; Figures 6G and H). Notably, the enriched lung cell population was not related to F4/80 positivity. The reduced R2 population was mainly associated with a reduction of CD68^−^F4/80^−^ double negative cells (data not shown). The enrichment of pulmonary CD68^+^ cells was further confirmed by immunohistochemical staining for CD68, which showed a significant increasing trend over time compared to sham control (Figure 6I).

### CD68^+^ F4/80^−^ CCR2^+^ and CD68^+^ F4/80^−^ CD206^+^cells increased in the ligated group

**Figure 7:**
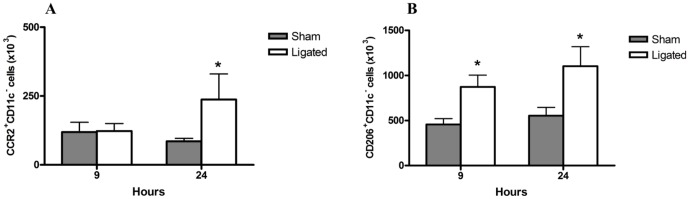
Phenotype profile CD68^+^ F4/80^−^ cells in the lungs following pancreatitis. Flow cytometry analysis of CCR2, CD11c (M1) and CD206 (M2) activation markers of lung macrophages gated for FSC/SSC (R1) and CD68^+^F4/80^−^. Significant enrichment of the number of CCR2^+^ CD11c^−^ (A) and CD206^+^ CD11c^−^ (B) cells in the lungs following pancreatitis (ligated) compared to sham controls. Bars show mean ±SEM, n = 8 per group. *P<0.05 versus sham control, by two-tailed Student *t*-test.

The phenotype of the enriched CD68^+^F4/80^−^ population was then determined based on the expression of CD11c, CD206 and CCR2. Ligated animals demonstrated a significant increase in the percentage of CD68^+^ F4/80^−^ CCR2^+^ cells in R1 at 24 h compared to the sham group (Figure 7A). The expression of CD206 marker was significantly enhanced in the CD68^+^ F4/80^−^ cell population in the R1 gate at both time points (Figure 7B). No significant changes in the CD11c^+^ cells in any of the groups or at any time point was seen. These data suggest that CD68^+^CD206^+^ and CD68^+^CCR2^+^ cells are significantly enriched and could contribute to the acute lung injury following acute pancreatitis.

## Discussion

The purpose of the current study was to investigate the dynamics of lung macrophages in acute lung injury induced in a murine ductal ligated acute pancreatitis model. Our data showed an enriched population of CD68^+^CCR2^+^ and CD68^+^CD206^+^ macrophages in the lungs in animals with acute pancreatitis.

Respiratory dysfunction is a major component of the multiple organ dysfunction syndrome in severe acute pancreatitis, and also represent a dominant contribution to mortality [12]. Previous studies have indicated that a variety of inflammatory factors, including inflammatory cells and cytokines, regulate the severity of pancreatitis and pancreatitis-associated lung injury [13]. Macrophages as one of the most versatile cells of the body have an important role in determining the severity of the acute lung injury [14]. However, the knowledge about responsible phenotypes for the injury and the resolution is still lacking.

In the present study, we used a murine biliary and pancreatic ductal ligation model resulting in acute pancreatitis. Ligation of the bile-pancreatic duct, proximal to the pancreas (common bile duct) and distal to the pancreas (at its junction with the duodenum), resulted in the development of edema, necrosis and an inflammatory response within the pancreas corresponding to the picture of acute pancreatitis. A pronounced both systemic and pulmonary inflammatory response, as well as morphological changes in the lungs were also seen.

Pancreatic ductal ligation causes activation of ERK MAP kinase, NF-κB and c-Jun, which are the key mediators for inflammation. This activation results in the production of different inflammatory mediators [10].

IL-6 is an important inflammatory mediator, which has been reported useful as a severity predictor in acute pancreatitis [15], [16]. The earliest difference between acute pancreatitis (BPD-ligated) and sham operated groups, was the early rise of IL-6 in plasma. This rise was not due to its production in the pancreas or lungs, since there was no difference in the level of IL-6 in tissue homogenates between acute pancreatitis (ligated) compared to sham operated animals at any time point studied. This implies that the difference in the IL-6 level could be due to production by e.g. circulating monocytes. This finding was similar to what previously was found in a model of ALI in association with acute ischemic kidney injury [17].

As with IL-6, TNF-α represents a major determinant of the systemic progression and end-organ damage such as acute lung injury in acute pancreatitis [18]. In this study, a significant rise in the acute phase plasma TNF-α level in BPD group was observed at 24 h compared to the sham operated group. Although utilizing a highly sensitive assay, the CVs at TNF-α levels below 5 pg/ml were relatively high and the low, although significant rise at the early 1 h was not considered biologically relevant.

The severity of pancreatitis and pancreatitis-associated ALI relates to the balance between pro-inflammatory and anti-inflammatory mediators. IL-10 is an anti-inflammatory cytokine that inhibits the release of pro-inflammatory cytokines from macrophages. Previous studies have shown a role of IL-10 in reducing the severity of acute pancreatitis and ALI [19], [20].

The inflammatory response in the pancreas and the lungs was studied by quantifying the levels of the main chemoattractant proteins for neutrophil (CXCL1) and macrophages (CCL2). The increased levels of the chemoattractants in both tissues were further investigated by analyzing the recruitment of both neutrophils and macrophages into pancreatic and lung tissue. The recruitment of neutrophils and macrophages in the pancreas followed the increased level of the corresponding chemoattractant. This was also noted in the lungs for neutrophils, but not for F4/80 positive macrophages. The findings are consistent with a previous study, in which F4/80 was used as a marker for detecting macrophages [21].

The CC chemokines, such as CCL2, macrophage inflammatory protein (MIP)-1α and RANTES are believed to primarily activate and recruit monocytes, whereas the CXC chemokines, such as CXCL1, preferentially tend to recruit neutrophils [22].

The CXCL1 increased levels in the pancreas and lungs in animals with acute pancreatitis compared to the sham operated group were not associated with a significant difference in the plasma levels between them. This finding along with the difference of the CXCL1 levels in the pancreas and lungs (almost four times less in the pancreas), indicate a local response in the lungs secreting CXCL1.

Considering the increased levels of the CCL2 in the lung tissue of the acute pancreatitis groups and the immunohistochemistry data for F4/80 antigen, the macrophage populations in the lungs were further analyzed by adding another cell marker (CD68). The data showed an enhanced population of macrophages that expressed CD68 and not F4/80. An increased expression of CD68 has previously been associated with macrophage activation [23]. Another possible explanation is the recruitment of these cells from the blood stream. The increase in the CD68^+^CCR2^+^ population in the acute pancreatitis group at 24 hours favors the recruitment of these cells via CCL2/CCR2 axis into the lungs. Although CD68 is routinely used as a histological marker of macrophage lineage cells, its specific function(s) in these cells remain undefined. CD68^+^ macrophages generated vasodilatory, angiogenic and proliferative growth factors in the hepatopulmonary syndrome in rats. They were recruited by the increased level of CCL2 to the lungs and their depletion prevented and reversed the pathological findings [24].

Cytokines and microbial products have profound effects on the mononuclear phagocytes and prime them towards their specialized polarization [25]. Macrophages activated through the alternative pathway express a repertoire of proteins involved in repair and healing, cell proliferation, and angiogenesis [26]. Upregulation of expression of CD206 (macrophage mannose receptor) distinguishes the alternative activation from the classical activation of macrophages. The increase in the CD206^+^ cells in the ligated group starting at 9 hours can be the result of different scenarios. This can be caused by an increase in CCL2 levels in lung tissue. CCL2 induces M2-type macrophage polarization in human peripheral blood by a significant increase in the mannose receptor (CD206) [27]. It has also previously been shown that CCL2 changes the ratio of M1/M2 macrophages in murine lungs towards a M2 phenotype [28]. Recruitment of CD68^+^ CD206^+^ cells into lung tissue can be the other explanation. Enrichment of alternatively activated macrophages occurs not only through coaxing their precursors from the bloodstream, but also by local proliferation of macrophages [29].The ratio of M1/M2 polarization changed at 24 hours towards a M1 phenotype by the increase in CD68^+^CCR2^+^ macrophages in the ligated group.

An ideal model for studying acute pancreatitis and its potentially associated multiple organ failure should resemble the human disease course, and it should be easily reproducible, have sufficient severity and still allow a time window long enough for potential intervention. Many of the available models are not fulfilling all these criteria. For this reason, different approaches with the ductal ligation model have been described in various animals [30]. The model we used in this study mimics acute biliary pancreatitis and avoids artificial drug usage, which may produce unwanted effects. The profound inflammatory response in the lungs makes this model relevant for study of the acute lung injury seen associated with acute pancreatitis. Other experimental models of acute pancreatitis are either not severe enough to induce lung injury, or do not resemble the course in the human clinical setting.

The development and resolution of lung injury is accompanied by dramatic changes not only in the numbers, but also the phenotypes of macrophages in the lungs. A better understanding of the regulation and function of different macrophage populations involved in acute pancreatitis-associated ALI can be helpful for establishing new modes of therapy.

There are questions remaining to be answered in future studies. It should be clarified weather the CD68^+^ F4/80^−^ population is recruited to the lungs, or is it the result of a change in the expression of the cell markers on resident macrophages. Next step will be focused on the function of these particular cells and their contribution to the inflammation.

In conclusion, our study demonstrates that there is substantial change in the heterogeneity of antigen expression by pulmonary macrophages in acute pancreatitis-associated ALI. This may provide future possibilities for exploiting the heterogeneity of macrophages as potential therapeutic targets.
